# NAT2 rs1495741 and anti-tuberculosis drug-induced liver injury in children: genetic association and risk prediction

**DOI:** 10.3389/fphar.2026.1810732

**Published:** 2026-05-08

**Authors:** Xin Huang, Jie Jiang, Li He, Yan Guo, Jianzhu Zhou, Hui Qiu, Haiyi Zhou, Xiaoyan Liu, Hanjie Yang, Chengxian Guo

**Affiliations:** 1 Center of Clinical Pharmacology, The Third Xiangya Hospital, Central South University, Changsha, Hunan, China; 2 Office of Clinical Trial Institution, Liuzhou Maternity and Child Healthcare Hospital, Liuzhou, Guangxi, China; 3 Department of Pediatrics, The Afffliated Changsha Central Hospital, Hengyang Medical School, University of South China, Changsha, China; 4 Department of Pediatrics, The Third Xiangya Hospital of Central South University, Changsha, China; 5 Hunan Normal University School of Medicine, Changsha, Hunan, China

**Keywords:** anti-tuberculosis drug-induced liver injury, causal mediation analysis, children, isoniazid, machine learning, NAT2 polymorphism

## Abstract

**Background:**

Anti-tuberculosis drug-induced liver injury (ATB-DILI) is a major adverse event in children receiving anti-tuberculosis therapy, but pediatric pharmacogenetic evidence remains limited.

**Methods:**

We studied 192 children receiving anti-tuberculosis treatment, including 31 with ATB-DILI and 161 without DILI. After quality control, 86 single-nucleotide polymorphisms were tested for association with ATB-DILI. Based on three available NAT2 variants, NAT2 diplotypes were inferred from the genotype data, and acetylator phenotypes were determined. The relationship of rs1495741 with 2-h plasma isoniazid concentration and ATB-DILI was further evaluated, and prediction models integrating genetic and clinical variables were developed.

**Results:**

After Bonferroni correction, only NAT2 rs1495741 remained significantly associated with ATB-DILI (*P* = 0.013). Under the recessive model, the AA genotype was associated with a 7.29-fold higher risk of ATB-DILI than the GG/AG genotypes (95% CI: 3.22–17.52; *P* < 0.001). In the NAT2 analysis, slow acetylator phenotypes were more frequent in the ATB-DILI group than in the non-DILI group (64.52% vs. 21.12%). Rs1495741 showed high concordance with inferred NAT2 acetylator phenotypes (Cohen’s kappa = 0.89, *P* < 0.001). Measured 2-h plasma isoniazid concentrations increased across rs1495741 genotypes, and the AA genotype was strongly associated with high isoniazid concentration (>6 μg/mL; OR = 11.23, 95% CI: 6.62–19.07; *P* < 0.001). The best predictive model achieved an AUC of 0.874.

**Conclusion:**

NAT2 rs1495741 was strongly associated with pediatric ATB-DILI risk and showed high concordance with inferred NAT2 acetylator phenotypes. This association was not significantly mediated by the measured single 2-h plasma isoniazid concentration in this dataset. Incorporating rs1495741 may improve risk stratification in children.

## 1 Introduction

Tuberculosis (TB), an infectious respiratory disease, remains one of the top ten causes of death worldwide (Zhou et al., 2025). Due to their still-developing immune systems, children exhibit not only higher susceptibility but also an elevated risk of severe disease progression (Marais, 2014). In the chemotherapy of tuberculosis, isoniazid (INH) serves as the cornerstone of first-line regimens owing to its potent bactericidal activity. However, anti-tuberculosis drug-induced liver injury (ATB-DILI) is the most common and serious adverse reaction during treatment, with its incidence in children varying widely (1.7%–26.8%) (Mehra et al., 2022; Aishatu et al., 2017; Mansukhani and Shah, 2012; Gafar et al., 2019; Bhatia et al., 2011; Abdusalomov et al., 2021). Mild cases may lead to treatment interruption or regimen modification, while severe instances can trigger acute liver failure or even death. Compared with adults, children differ significantly in the activity levels of hepatic metabolic enzymes, drug clearance capacity, and tolerance thresholds for hepatotoxicity. (Kearns et al., 2003; Molleston et al., 2011). Consequently, directly applying adult risk assessment frameworks often lacks precision in pediatric populations.

INH metabolism in the body primarily relies on the hepatic N-acetyltransferase 2 (NAT2) (Richardson et al., 2019). This enzyme is encoded by the NAT2 gene, which exhibits high polymorphism. Based on genetic variations, individuals can be classified into rapid, intermediate, and slow acetylator phenotypes (Perwitasari et al., 2015). Previous studies in adults have extensively confirmed that NAT2 polymorphisms dictate the rate of INH biotransformation, with slow acetylators exhibiting reduced drug clearance. However, in pediatric populations, the extent to which the association between NAT2 variation and hepatotoxicity is explained by measured parent-drug exposure remains unclear, particularly when exposure is represented by a single post-dose concentration. This nuanced mechanism remains largely unexplored in pediatric populations. Importantly, from a developmental pharmacology perspective, the phenotypic activity of NAT2 has been shown to be present and functionally mature from birth, suggesting that genetic polymorphisms could theoretically influence drug metabolism and toxicity risks in children as early as infancy (Zhu et al., 2012). Nevertheless, pediatric studies have largely focused on isolated genetic association analyses rather than jointly evaluating genotype, measured exposure, and clinical outcomes.

In addition, while conventional statistical methods have inherent limitations in modeling complex non-linear relationships and integrating high-dimensional data, machine learning (ML) offers a powerful tool for capturing such complexity and improving risk prediction (Sidey-Gibbons and Sidey-Gibbons, 2019; Topol, 2019). Beyond prediction, mediation analysis can be used to assess whether observed genetic associations with hepatotoxicity are statistically mediated, in part, by measured exposure variables. Importantly, such analyses do not by themselves establish a direct biological mechanism.

Accordingly, this study establishes a prospective cohort of pediatric tuberculosis patients in China with the following objectives (Zhou et al., 2025): to systematically screen key single nucleotide polymorphisms (SNPs) potentially associated with ATB-DILI in children, a population less frequently represented in pharmacogenetic studies (Marais, 2014); to explore, using causal mediation analysis, whether the association between genetic variation and hepatotoxicity may be statistically mediated, in part, by measured isoniazid plasma concentration in pediatric populations; and (Mehra et al., 2022) to develop and evaluate machine learning-based predictive models integrating multimodal data, and to assess whether incorporating genetic markers could offer additional value for risk prediction. By addressing these aspects, this study aims to identify children at increased risk of ATB-DILI, thereby informing personalized monitoring and preventive strategies in pediatric tuberculosis treatment.

## 2 Materials and methods

### 2.1 Study design and study subjects

This study included pediatric patients diagnosed with tuberculosis at Changsha Central Hospital between January 2023 and December 2024, all of whom received INH-containing regimens. Inclusion criteria were (Zhou et al., 2025): meeting the diagnostic criteria specified in the National Health Industry Standard of the People’s Republic of China: Diagnostic Criteria for Pulmonary Tuberculosis (Marais, 2014); age <18 years; and (Mehra et al., 2022) ability to complete the required examinations and follow-up. A total of 192 eligible children were finally enrolled. The study protocol strictly adhered to the principles of the Declaration of Helsinki and was approved by the Ethics Review Committee of Changsha Central Hospital (Approval No.: Medical Review 2025–009).

**FIGURE 1:**
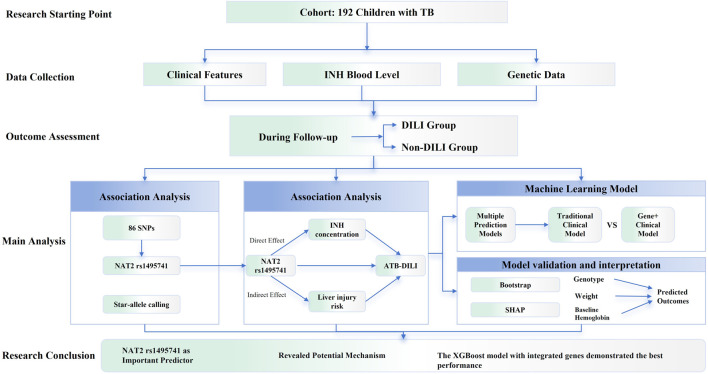
Schematic of the study workflow and analytical framework. After initial screening of 277 cases, 85 were excluded based on the exclusion criteria, resulting in a final total sample size of N = 192 for statistical analysis.

The overall workflow and analytical framework are illustrated in Figure 1. The study consisted of three phases (Zhou et al., 2025): Multimodal Data Collection, covering clinical characteristics, therapeutic drug monitoring, and genetic data (Marais, 2014); Outcome Follow-up, with participants stratified based on liver function monitoring results; and (Mehra et al., 2022) Comprehensive Analysis, integrating association analysis, exploration of causal mediation effects, and predictive modeling using machine learning.

### 2.2 Data collection and outcome definition

This study systematically collected baseline demographic characteristics, clinical-pathological data, anti-tuberculosis treatment regimens (drug dosage, frequency), comorbidities, and concurrent medications. For outcome definition, liver function parameters were closely monitored during treatment according to the routine clinical follow-up schedule: at baseline (prior to treatment initiation) and monthly thereafter until the end of therapy. Additional tests were performed promptly if patients presented with symptoms suggestive of liver injury (e.g., nausea, vomiting, jaundice). ATB-DILI was diagnosed according to established guidelines, defined as (Zhou et al., 2025): serum alanine aminotransferase (ALT) ≥ 3 times the upper limit of normal (ULN) and/or total bilirubin (TBIL) ≥ 2 times ULN; or (Marais, 2014) concurrent elevation of aspartate aminotransferase (AST), ALT, and TBIL, with at least one parameter ≥2 times ULN. Based on the occurrence of DILI, patients were categorized into the ATB-DILI group or the non-DILI group.

### 2.3 Genotype and therapeutic drug monitoring

A total of 97 SNP loci related to anti-tuberculosis drug metabolism, transport, and hepatotoxicity were selected by searching the ClinPGx database and reviewing relevant literature (Supplementary Table S1). The selection was based on a multi-criteria framework prioritizing SNPs with (Zhou et al., 2025): established clinical annotations in the ClinPGx database for isoniazid/rifampicin pharmacokinetics or toxicity (Marais, 2014); reported associations with ATB-DILI in previous meta-analyses; and (Mehra et al., 2022) coverage of key pathways (e.g., NAT2 acetylation, CYP-mediated metabolism, oxidative stress response). Genomic DNA was extracted from peripheral venous blood samples, and high-throughput genotyping was performed using the MassARRAY platform. Strict quality control was conducted with PLINK 1.9 software, retaining only SNPs meeting all of the following criteria: genotype missing rate ≤10%, consistent with Hardy–Weinberg equilibrium (HWE, *P* > 0.05), and minor allele frequency (MAF) ≥ 0.01. After patients had continuously taken isoniazid for at least 5 days (to reach steady-state concentration), venous blood samples were collected 2 h after the morning dose (Thee et al., 2011). A standardized 2-h post-dose plasma sample was collected for isoniazid concentration measurement, which was determined by high-performance liquid chromatography. Among the genotyped loci, rs1495741 showed a statistically significant association with ATB-DILI. To contextualize this finding within a NAT2 pharmacogenetic framework, we leveraged the three available genotyped NAT2 variants (rs1799930, rs1801280, and rs1799931) to perform star-allele calling according to PharmVar nomenclature (Papanikolaou et al., 2026), restricting analysis to resolvable common alleles. These three variants capture the major resolvable common slow-acetylator-associated NAT2 alleles and were used for star-allele assignment according to a PharmVar-aligned rule-based framework limited to the alleles resolvable from the available data (*4, *5, *6, and *7) (Papanikolaou et al., 2026). Based on these allele calls, NAT2 diplotypes were reconstructed and translated into acetylator phenotypes (rapid, intermediate, and slow) using prespecified phenotype translation rules. As a supplementary analysis, concordance between rs1495741 genotype categories and the diplotype-derived NAT2 acetylator phenotypes was also evaluated.

### 2.4 Statistical analysis

All statistical analyses were performed in R (v4.4.2; RStudio) and Python (v3.11.4). Continuous variables were compared using Student’s t-test or the Mann–Whitney U test, as appropriate, and categorical variables using the Chi-squared test or Fisher’s exact test. Genetic associations with ATB-DILI were evaluated by logistic regression in PLINK 1.9, with odds ratios (ORs) and 95% confidence intervals (CIs) reported; Bonferroni-corrected P < 0.05 was considered statistically significant. Using the three available NAT2 variants (rs1799930, rs1801280, and rs1799931), NAT2 star alleles were assigned according to a prespecified PharmVar-aligned rule-based framework limited to the common resolvable alleles (*4, *5, *6, and *7). Diplotypes were then reconstructed and translated into rapid, intermediate, and slow acetylator phenotypes. Concordance between rs1495741 genotype categories and diplotype-derived acetylator phenotypes was assessed as a supplementary analysis using Cohen’s kappa coefficient. To evaluate whether the association between rs1495741 and ATB-DILI was statistically mediated by measured 2-h plasma isoniazid concentration, causal mediation analysis was performed using the R package mediation. Rs1495741 genotype was treated as the independent variable, measured 2-h plasma isoniazid concentration as the mediator, and ATB-DILI as the dependent variable. The average causal mediation effect (ACME) and average direct effect (ADE) were estimated using non-parametric bootstrapping with 1,000 resamples.

### 2.5 Model development and evaluation

#### 2.5.1 Data preprocessing and feature selection

As the majority of features exhibited low missing rates (<10%), mean imputation was applied to continuous variables before model training; categorical variables contained no missing data. This approach was deemed appropriate given the limited missingness and sample size constraints. Subsequently, Random Forest was employed for feature dimensionality reduction, retaining variables with non-zero feature importance and weights greater than 4%. The final feature combinations for model training were determined by integrating these results with clinical relevance. To address the class imbalance in the dataset, the Synthetic Minority Over-sampling Technique (SMOTE) was applied within the training set for data balancing. All subsequent modeling steps, including feature selection, class rebalancing with SMOTE, and hyperparameter tuning, were performed within the cross-validation folds to prevent information leakage.

#### 2.5.2 Model training

The dataset was randomly split into a training set and a test set at a ratio of 7:3. Seven machine learning algorithms were included for comparison: XGBoost, LightGBM, Logistic Regression, Random Forest (RF), Gradient Boosting Decision Tree (GBDT), Multilayer Perceptron (MLP), and AdaBoost. In the training set, a five-fold cross-validation combined with grid search was employed for hyperparameter tuning to prevent overfitting. To systematically evaluate the incremental predictive value of clinical characteristics, genetic factors, and drug exposure for DILI, four feature combination models were designed: Model A: Included only routine clinical features; Model B: Model A+ INH concentration; Model C: Model A+ genotype data; Model D: Model A+ INH concentration + genotype data.

#### 2.5.3 Model validation and interpretation

Model performance was comprehensively evaluated using the area under the receiver operating characteristic curve, accuracy, and specificity. To validate the robustness of the models, 1000-time bootstrap internal validation was performed. Finally, the SHapley Additive exPlanations (SHAP) method was introduced to interpret the best-performing model, quantifying the contribution of each feature to individual predictions and enabling visual model interpretation.

## 3 Results

### 3.1 Baseline characteristics

A total of 192 pediatric patients were included, of whom 31 cases (16.15%) were classified into the liver injury group and 161 cases into the non-liver injury group. As shown in Table 1, compared with the non-liver injury group, patients in the liver injury group had a significantly lower mean body weight (*P* = 0.008), a higher initial isoniazid (INH) dosage (*P* = 0.021), a markedly elevated serum concentration of INH (*P* < 0.001), and were more frequently treated concurrently with Prednisone (*P* = 0.039).

**TABLE 1:** Demographic and clinical characteristics of patients with ATB-DILI and without ATB-DILI.

Characteristics	Non-DILI	ATB-DILI	*P-value*
Number of patients (n,%)	161 (83.85)	31 (16.15)	NA
Sex (n,%)			
Male	81 (50.31)	19 (61.29)	0.355^a^
Age (years)	11.58 ± 4.58	9.69 ± 5.11	0.062
Weight (kg)	40.87 ± 16.99	32.22 ± 15.82	0.008
Allergy (n,%)	12 (7.45)	1 (3.23)	0.640^b^
Confirmed in-hospital diagnosis (n,%)	129 (80.12)	21 (67.74)	0.197
Initial hospitalization duration (days)	13.27 ± 7.24	20.16 ± 9.18	< 0.001
Presence of extrapulmonary TB	25 (15.53)	8 (25.81)	0.259^a^
Complication (n,%)			
Liver and gallbladder diseases	5 (3.11)	1 (3.23)	1.000^b^
Undernourishment	27 (16.77)	9 (29.03)	0.177^a^
Hypoproteinemia	7 (4.35)	2 (6.45)	0.965^b^
Anemic	15 (9.32)	7 (22.58)	0.069^b^
Combination of drugs (n,%)			
Azithromycin	35 (21.74)	6 (19.35)	0.954^a^
Prednisone	24 (14.91)	10 (32.26)	0.039^a^
Ceftriaxone	9 (5.59)	3 (9.68)	0.648^b^
Ibuprofen	9 (5.59)	4 (12.90)	0.274^b^
Initial dose of isoniazid (mg/kg)	7.72 ± 2.57	9.11 ± 3.01	0.021
Initial dose of rifampicin (mg/kg)	10.43 ± 2.08	10.85 ± 1.81	0.258
Concentration of isoniazid (μg/mL)	6.47 ± 3.20	9.39 ± 3.89	< 0.001
Concentration of rifampicin (μg/mL)	9.01 ± 4.45	9.45 ± 4.89	0.475
Baseline laboratory values (Mean ± SD)			
ALB (g/L)	39.02 ± 4.48	37.71 ± 4.78	0.168
TBIL (μmol/L)	8.49 ± 4.55	8.15 ± 4.50	0.694
DBIL (μmol/L)	3.00 ± 1.78	3.17 ± 2.30	0.691
ALT (u/L)	17.37 ± 10.67	48.12 ± 89.51	0.066
AST (u/L)	24.52 ± 10.95	47.81 ± 69.80	0.074
TBA (μmol/L)	6.22 ± 7.86	6.01 ± 4.21	0.832
PA (mg/L)	192.64 ± 48.11	180.97 ± 41.18	0.167
Cr (μmol/L)	43.39 ± 15.15	38.46 ± 11.34	0.042
UREA (mg/dL)	5.81 ± 19.04	3.77 ± 1.31	0.187
UA (μmol/L)	320.28 ± 116.89	288.80 ± 90.56	0.098
WBC (10^9^/L)	7.87 ± 3.17	8.58 ± 4.34	0.393
RBC (10^12^/L)	6.31 ± 12.82	4.94 ± 0.94	0.180
HGB (g/L)	124.03 ± 22.68	116.16 ± 13.39	0.011
PLT (10^9^/L)	319.00 ± 94.67	376.57 ± 155.07	0.054
Most recent laboratory test value (Mean ± SD)			
ALB (g/L)	40.77 ± 4.32	39.99 ± 2.06	0.126
PA (mg/L)	215.73 ± 31.04	216.58 ± 3.98	0.739
Cr (μmol/L)	46.48 ± 13.76	40.43 ± 11.87	0.015
UREA (mg/dL)	4.07 ± 2.43	4.67 ± 4.06	0.431
UA (μmol/L)	469.97 ± 177.50	376.34 ± 134.66	0.002

WBC, white blood cell count; RBC, red blood cell count; HGB, hemoglobin; PLT, platelet; ALB, albumin; ALT, alanine aminotransferase; AST, aspartate aminotransferase; TBIL, total bilirubin; DBIL, direct bilirubin; TBA, total bile acid; Cr, creatinine; UREA, urea; UA, uric acid; PA, prealbumin.

^a^ Chi-square test.

^b^ Fisher’s exact test.

### 3.2 Association of NAT2 rs1495741 with ATB-DILI

After quality control, 86 SNPs were included in the subsequent correlation analysis. Following Bonferroni correction, only the rs1495741 locus in the NAT2 gene showed a statistically significant association with the risk of ATB-DILI in children (*P* = 0.013). Under the recessive genetic model, patients carrying the AA homozygous genotype had a 7.29-fold increased risk of developing ATB-DILI compared to those carrying the GG or AG genotypes (95% CI: 3.22–17.52, *P* < 0.001) (Supplementary Table S2). The genotype counts of rs1495741 in the ATB-DILI and non-ATB-DILI groups are shown in Supplementary Table S3.

### 3.3 NAT2 star-allele, diplotype, and acetylator phenotype analysis

For placing the rs1495741 association within a PharmVar-aligned NAT2 pharmacogenetic framework, we assigned NAT2 star alleles using the three available genotyped NAT2 variants in this study (rs1799930, rs1801280, and rs1799931), following a rule-based classification of the common resolvable alleles (*4, *5, *6, and *7) (Supplementary Table S4). Based on these allele assignments, NAT2 diplotypes were reconstructed and further translated into acetylator phenotypes (rapid, intermediate, and slow).

The distribution of NAT2 diplotypes in children with and without ATB-DILI is shown in Table 2. Across the cohort, the most frequent diplotypes were *4/*6 (27.08%), *4/*4 (24.48%), and *4/*7 (17.71%). Diplotypes classified as slow acetylation patterns were more common in the DILI group than in the non-DILI group, whereas the fully rapid diplotype was less frequent among DILI cases.

**TABLE 2:** Distribution of NAT2 diplotypes derived from available genotyped NAT2 variants in children with and without ATB-DILI.

NAT2 diplotype	Non-DILI (n = 161)	DILI (n = 31)	Total (n = 192)
*4/*6	48 (29.81%)	4 (12.90%)	52 (27.08%)
*4/*4	43 (26.71%)	4 (12.90%)	47 (24.48%)
*4/*7	32 (19.88%)	2 (6.46%)	34 (17.71%)
*6/*7	18 (11.18%)	8 (25.81%)	26 (13.54%)
*6/*6	9 (5.59%)	6 (19.35%)	15 (7.81%)
*4/*5	4 (2.49%)	1 (3.22%)	5 (2.61%)
*5/*7	3 (1.86%)	2 (6.46%)	5 (2.61%)
*5/*6	1 (0.62%)	3 (9.68%)	4 (2.08%)
*7/*7	3 (1.86%)	1 (3.22%)	4 (2.08%)

At the phenotype level, the distribution of diplotype-derived NAT2 acetylator phenotypes differed between groups (Table 3). Slow acetylators accounted for 64.52% of children with ATB-DILI, compared with 21.12% of those without DILI. In contrast, rapid acetylators accounted for 12.90% of DILI cases and 26.71% of non-DILI controls, while intermediate acetylators accounted for 22.58% and 52.17%, respectively. These findings support the relevance of NAT2-related acetylation status to susceptibility to ATB-DILI in children.

**TABLE 3:** Distribution of NAT2 acetylator phenotypes in the non-DILI and DILI groups.

NAT2 phenotype	non-DILI (n = 161)	DILI (n = 31)	Total (n = 192)
Rapid	43 (26.71%)	4 (12.90%)	47 (24.48%)
Intermediate	84 (52.17%)	7 (22.58%)	91 (47.40%)
Slow	34 (21.12%)	20 (64.52%)	54 (28.12%)

We further compared rs1495741 genotype categories with the diplotype-derived NAT2 acetylator phenotypes and observed a high degree of concordance (Cohen’s kappa = 0.89, P < 0.001; Supplementary Table S5), supporting the interpretability of rs1495741 as a marker of NAT2 acetylation status in this cohort.

### 3.4 Association of rs1495741 genotype with measured 2-h plasma INH concentration

**FIGURE 2:**
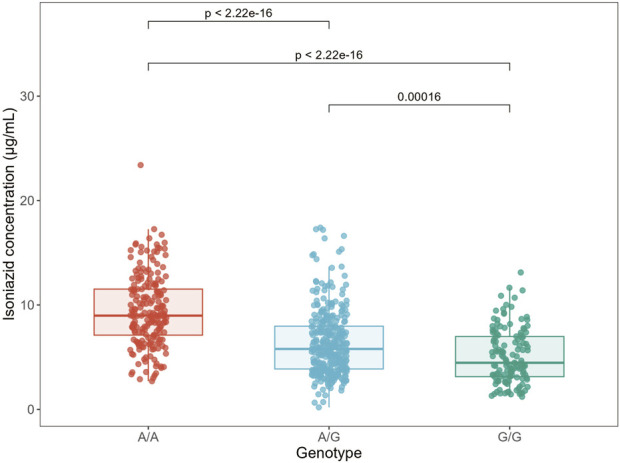
Association between NAT2 rs1495741 genotype and plasma concentration of Isoniazid. The boxplot illustrates the distribution of drug concentrations among patients with the GG (n = 43), AG (n = 92), and AA (n = 57) genotypes.

To further characterize the pharmacokinetic relevance of rs1495741, we analyzed the relationship between genotype and measured 2-h plasma isoniazid concentration. Mean INH concentrations exhibited a stepwise increase across the three genotype groups (GG, AG, and AA), reaching 5.12 ± 2.61 μg/mL, 6.32 ± 3.13 μg/mL, and 9.41 ± 3.40 μg/mL, respectively; the overall difference was statistically significant (*P* < 0.05) (Figure 2). Further analysis showed that, compared with patients carrying the GG genotype, those with the AA genotype had a markedly increased likelihood of having high INH concentrations (>6 μg/mL), with an OR of 11.23 (95% CI: 6.62–19.07, *P* < 0.001). These findings indicate that rs1495741 is strongly associated with measured isoniazid exposure in this cohort and is consistent with rs1495741 serving as a marker of reduced NAT2 acetylation capacity in this cohort.

### 3.5 Mediation effect analysis

**FIGURE 3:**
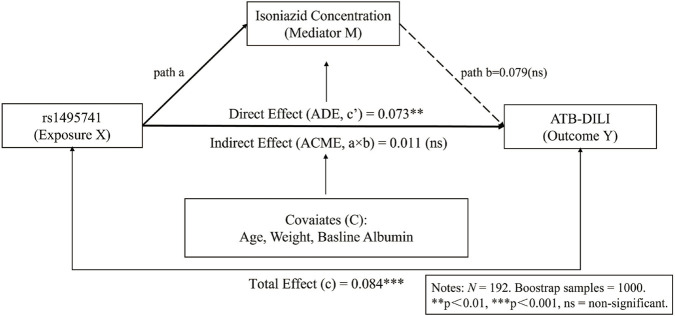
Causal mediation analysis of the association between NAT2 rs1495741 genotype, isoniazid concentration, and ATB-DILI. ATB-DILI, anti-tuberculosis drug-induced liver injury.

To investigate whether the effect of rs1495741 on liver injury was mediated through the measured isoniazid concentration, we performed a causal mediation analysis (Figure 3; Table 4). Although univariate analysis showed higher 2-h isoniazid concentrations in the DILI group, the average causal mediation effect was not statistically significant (ACME *P* = 0.398). In contrast, the average direct effect remained significant (ADE estimate = 0.073, *P* = 0.004). As shown in Figure 3, the indirect effect was small and not statistically significant, whereas the direct effect remained evident. These findings indicate that the observed association was not significantly mediated by the measured single 2-h parent-drug concentration in this dataset. Because no longitudinal pharmacokinetic or metabolite measurements were available, no further mechanistic inference should be drawn from this analysis.

**TABLE 4:** Mediation analysis of the effect of rs1495741 on Liver Injury mediated by Isoniazid Concentration.

Effect type	Estimate	95% CI lower	95% CI upper	*P-value*
Total effect	0.084	0.046	0.111	< 0.001
ADE	0.073	0.024	0.104	0.004
ACME	0.011	−0.013	0.045	0.398

ACME (Average Causal Mediation Effect); ADE (Average Direct Effect).

### 3.6 Integrated genetic-clinical model predicts ATB-DILI

After feature selection, the clinical variables finally included in the model were Baseline Alanine Aminotransferase, Initial Dose of Rifampicin, weight, Baseline Hemoglobin, Initial Dose of Isoniazid, Baseline Platelet, Baseline Aspartate Aminotransferase, among others (Supplementary Figure S1). We trained 28 models based on four feature combinations. The results of internal validation *via* Bootstrap (Supplementary Table S6) showed that models incorporating both the rs1495741 genotype and clinical variables generally outperformed those using clinical variables alone.

**FIGURE 4:**
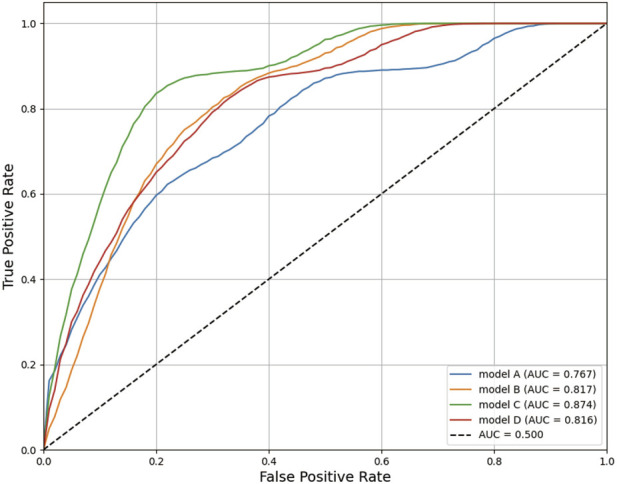
Comparison of ROC curves for models predicting ATB-DILI. ROC, Receiver Operating Characteristic; ATB-DILI, anti-tuberculosis drug-induced liver injury.

Among all models, those trained using the XGBoost algorithm demonstrated relatively better overall performance (Table 5). The ROC curves of the four models are shown in Figure 4, with the model integrating clinical variables and the rs1495741 genotype exhibiting the best performance. The model achieved an AUC of 0.874 (95% CI 0.870–0.877) in internal validation, with an accuracy of 0.843 and specificity of 0.917. Given the relatively small sample size and class imbalance, we focused on AUC as the primary performance metric, as it provides a more comprehensive assessment of model discrimination across all classification thresholds.

**TABLE 5:** Performance results of all models following bootstrap validation.

Model	Algorithm	Bootstrap validation		
		AUC (95%CI)	Accuracy	Specificity
Model A	XGBoost	0.767 (0.761–0.773)	0.844	0.918
Model B	XGBoost	0.817 (0.813–0.821)	0.811	0.898
Model C	XGBoost	0.874 (0.870–0.877)	0.843	0.917
Model D	XGBoost	0.816 (0.811–0.820)	0.826	0.919
Model A	LightGBM	0.843 (0.839–0.846)	0.739	0.752
Model B	LightGBM	0.799 (0.795–0.803)	0.740	0.775
Model C	LightGBM	0.832 (0.827–0.837)	0.745	0.738
Model D	LightGBM	0.855 (0.852–0.859)	0.757	0.754
Model A	RF	0.842 (0.838–0.846)	0.830	0.900
Model B	RF	0.837 (0.834–0.841)	0.812	0.880
Model C	RF	0.855 (0.851–0.859)	0.845	0.879
Model D	RF	0.829 (0.825–0.834)	0.859	0.917
Model A	GBDT	0.735 (0.730–0.741)	0.792	0.856
Model B	GBDT	0.764 (0.760–0.768)	0.778	0.858
Model C	GBDT	0.819 (0.814–0.823)	0.791	0.876
Model D	GBDT	0.796 (0.791–0.801)	0.809	0.897
Model A	AdaBoost	0.661 (0.655–0.668)	0.757	0.796
Model B	AdaBoost	0.666 (0.660–0.673)	0.742	0.838
Model C	AdaBoost	0.793 (0.787–0.799)	0.808	0.835
Model D	AdaBoost	0.668 (0.662–0.674)	0.758	0.857
Model A	MLP	0.778 (0.772–0.783)	0.809	0.878
Model B	MLP	0.795 (0.790–0.800)	0.845	0.898
Model C	MLP	0.843 (0.840–0.847)	0.777	0.839
Model D	MLP	0.818 (0.814–0.823)	0.827	0.897
Model A	LR	0.795 (0.791–0.800)	0.691	0.675
Model B	LR	0.805 (0.801–0.809)	0.693	0.679
Model C	LR	0.860 (0.856–0.864)	0.741	0.714
Model D	LR	0.830 (0.826–0.834)	0.757	0.734

Model A: incorporating routine clinical features only.

Model B: Model A+ isoniazid concentration.

Model C: Model A+ genotype data.

Model D: Model A+ isoniazid concentration + genotype data.

AUC, area under the curve; XGBoost, eXtreme Gradient Boosting; LightGBM, light gradient boosting machine; RF, random forest; GBDT, gradient boosting decision tree; AdaBoost, Adaptive Boosting; LR, logistic regression; MLP, multilayer perceptron.

### 3.7 SHAP analysis results

**FIGURE 5:**
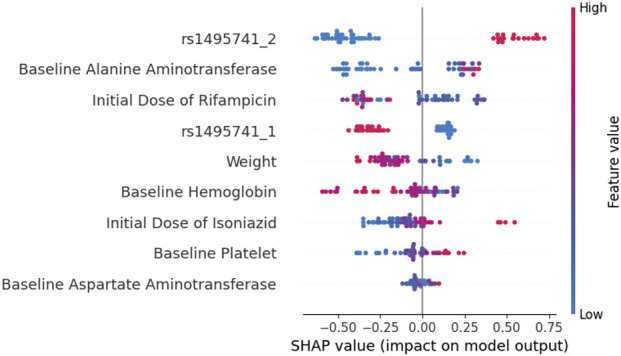
Interpretation of the ATB-DILI prediction model using SHAP analysis. ATB-DILI, anti-tuberculosis drug-induced liver injury; SHAP, SHapley Additive exPlanations. Each point represents a single sample, with color indicating the feature value (red for high, blue for low). The X-axis represents the SHAP value (positive values indicate a positive contribution to the risk prediction). Features are ranked in descending order of importance from top to bottom. The original rs1495741 variable was coded as 0, 1, and two and was transformed into two dummy variables for model input: rs1495741_1 indicating rs1495741 = 1 and rs1495741_2 indicating rs1495741 = 2, with rs1495741 = 0 as the reference category.

The SHAP feature importance ranking indicated that the NAT2 rs1495741 mutant homozygous genotype was the primary predictor (Figure 5). As shown in Figure 5, individuals carrying the risk genotype (high feature values, typically represented in red) generally exhibited higher positive SHAP values, suggesting a strong association with increased DILI risk. Conversely, individuals with the protective genotype (low feature values, typically represented in blue) mostly displayed negative SHAP values, indicating a protective effect. Additionally, Baseline Alanine Aminotransferase, Initial Dose of Rifampicin, weight, Baseline Hemoglobin, Initial Dose of Isoniazid, Baseline Platelet, and Baseline Aspartate Aminotransferase also contributed substantially to the model’s decision-making.

## 4 Discussion

This study investigated the association between NAT2-related genetic variation and pediatric ATB-DILI. Using a NAT2 classification based on the three available NAT2 variants, we observed a marked enrichment of slow acetylator phenotypes among DILI cases, and rs1495741 showed good concordance with this phenotype classification. In addition, rs1495741 was significantly associated with ATB-DILI risk, and incorporation of genotype information improved risk stratification in the predictive analysis. Taken together, these findings suggest that NAT2-related genetic variation may contribute meaningfully to susceptibility to ATB-DILI in children and may have potential value in individualized risk assessment.

A key finding of this study was the strong relationship between NAT2-related variation and DILI susceptibility. Children carrying the rs1495741 A A genotype had a markedly increased risk of developing DILI, with an OR greater than 7. This effect size appears larger than the 2.8-fold risk reported in adult studies (Suvichapanich et al., 2018), although this difference should be interpreted cautiously because of differences in study populations, sample size, and study design. Importantly, the NAT2 analysis also showed a substantial enrichment of slow acetylator phenotypes among DILI cases, which is consistent with the broader literature identifying NAT2 slow-acetylator status as an important determinant of isoniazid-related hepatotoxicity (Tavkar et al., 2025; Mahajan and Tyagi, 2024). The concordance analysis further showed that rs1495741 was in good agreement with the diplotype-derived acetylator phenotype, supporting its use as a practical surrogate marker of NAT2 acetylation status in this cohort. Because NAT2 activity is a major determinant of isoniazid disposition, these results support an association between phenotype-defined reduced acetylation capacity and increased susceptibility to ATB-DILI in children. Although NAT2 expression reaches adult levels relatively early after birth (Hines, 2008), developmental differences in hepatic defense and detoxification capacity have been described in pediatric populations (Kearns et al., 2003). Our data do not directly evaluate these processes, but such age-related factors may partly contribute to the magnitude of the observed genetic association and warrant further study.

Another notable finding was that the rs1495741 A A genotype was associated with significantly higher plasma INH concentrations. However, the mediation analysis based on the measured 2-h parent-drug concentration did not provide evidence that this single concentration measure fully explained the observed association with hepatotoxicity. This result is broadly consistent with previous literature indicating that NAT2 genotype influences isoniazid pharmacokinetics, whereas the relationship between INH exposure and DILI may not be explained solely by a single parent-drug measurement (Mahajan and Tyagi, 2024). Because our study included only one post-dose plasma measurement and did not quantify downstream metabolites, the mediation analysis should be interpreted as a statistical exploration rather than mechanistic evidence. Prior studies have suggested that alternative metabolic pathways and downstream metabolites, including hydrazine-related intermediates, may also be relevant to toxicity (Ulanova et al., 2024; Metushi et al., 2011). However, these possibilities could not be evaluated directly in the present study. Overall, our findings suggest that the observed association is not fully captured by a single time-point parent-drug measurement. In this context, genotype-based stratification may represent a useful adjunct to therapeutic monitoring strategies, and future studies incorporating longitudinal pharmacokinetic data and metabolite measurements will be important for clarifying these relationships.

The SHAP-based interpretability analysis of the machine learning model (Lundberg et al., 2020) provided additional support for the importance of rs1495741 in risk prediction. Among all included features, the mutant homozygous genotype of rs1495741 contributed most strongly to model output, and individuals carrying the risk genotype tended to show positive SHAP values corresponding to a higher predicted probability of DILI. This observation is consistent with established pharmacogenetic knowledge that NAT2 polymorphisms influence the acetylation rate of isoniazid (Khan et al., 2019). The rs1495741 A A genotype, which showed high concordance with the slow-acetylator classification in this cohort, was associated with increased hepatotoxicity risk and may reflect reduced acetylation capacity. The predictive importance of this variable should therefore be interpreted primarily in terms of risk stratification, rather than as evidence for a specific biological pathway.

Comparison of the four feature combinations further suggested the importance of genotyping in the predictive framework. Incorporating isoniazid concentration into the model did not improve performance as expected. In the best-performing XGBoost algorithm, the model including both genotype and blood concentration (Model D) performed slightly less well than the model including genotype alone (Model C). This finding does not imply that drug concentration is irrelevant to the pathogenesis of DILI. Rather, it suggests that, for prediction purposes, genotype may serve as a more stable upstream indicator of metabolic capacity, whereas a single concentration measurement may be more vulnerable to variability related to sampling time, short-term exposure fluctuation, and assay error. From this perspective, genotype and concentration may capture overlapping but non-identical aspects of risk, and the incremental predictive value of a single time-point concentration measurement may therefore be limited.

Several limitations of this study should be acknowledged. First, the sample size, particularly the number of DILI events, was limited. Although the study had sufficient power to detect the strong association observed for rs1495741, it may have been underpowered to identify weaker genetic associations or to support robust subgroup analyses. Therefore, the findings related to the machine learning models and the mediation analysis should be considered exploratory and require confirmation in larger, independent pediatric cohorts. Second, because only a limited number of NAT2 variants were available in the present dataset, the related findings require further validation in larger studies with more comprehensive genetic data. Third, this was a single-center study without an external validation cohort. Despite the use of internal validation procedures, overfitting remains a concern, and the generalizability of the model across different regions and ethnic populations requires further assessment in independent multicenter studies. Fourth, only a single time-point concentration measurement was available, which may not adequately reflect cumulative exposure or the dynamic pharmacokinetic processes relevant to liver injury. More comprehensive pharmacokinetic studies in children are therefore needed. Fifth, because downstream metabolites were not measured, no mechanistic inference can be made from the present data regarding how NAT2-related variation may translate into hepatotoxicity. Future studies integrating longitudinal pharmacokinetic sampling, metabolite profiling, and functional experimental approaches may help clarify these relationships. Finally, because the frequency and structure of NAT2 alleles vary across populations (Peretolchina et al., 2024), the applicability of the present findings to other ethnic groups should be interpreted with caution.

## 5 Conclusion

In this pediatric cohort, NAT2 rs1495741 was significantly associated with susceptibility to ATB-DILI, and the AA genotype was linked to both a markedly increased risk of liver injury and higher measured 2-h plasma isoniazid concentrations. Within the NAT2 pharmacogenetic framework based on the available NAT2 variants, slow acetylator phenotypes were substantially enriched among children with ATB-DILI, and rs1495741 showed high concordance with diplotype-derived acetylator phenotypes. In addition, incorporating rs1495741 into clinical prediction models improved discrimination for ATB-DILI risk. These findings support the potential value of NAT2-related genetic variation, particularly rs1495741, in pediatric risk stratification for ATB-DILI. Further validation in larger, independent cohorts with more comprehensive pharmacokinetic and genetic data is warranted.

## Data Availability

All derived data relevant to this study are contained within the article/supplementary material. Due to privacy concerns and informed consent restrictions, the original genotype data cannot be made publicly available.

## Supplementary material

SUPPLEMENTARY FIGURE S1 Variable importance plot from the feature selection process.
