# Supplementary figures and images for: NAT2 rs1495741 and anti-tuberculosis drug-induced liver injury in children: genetic association and risk prediction
Source: Front Pharmacol. 2026 May 8;17:1810732. doi: 10.3389/fphar.2026.1810732 (PMC13195251; doi:10.3389/fphar.2026.1810732)

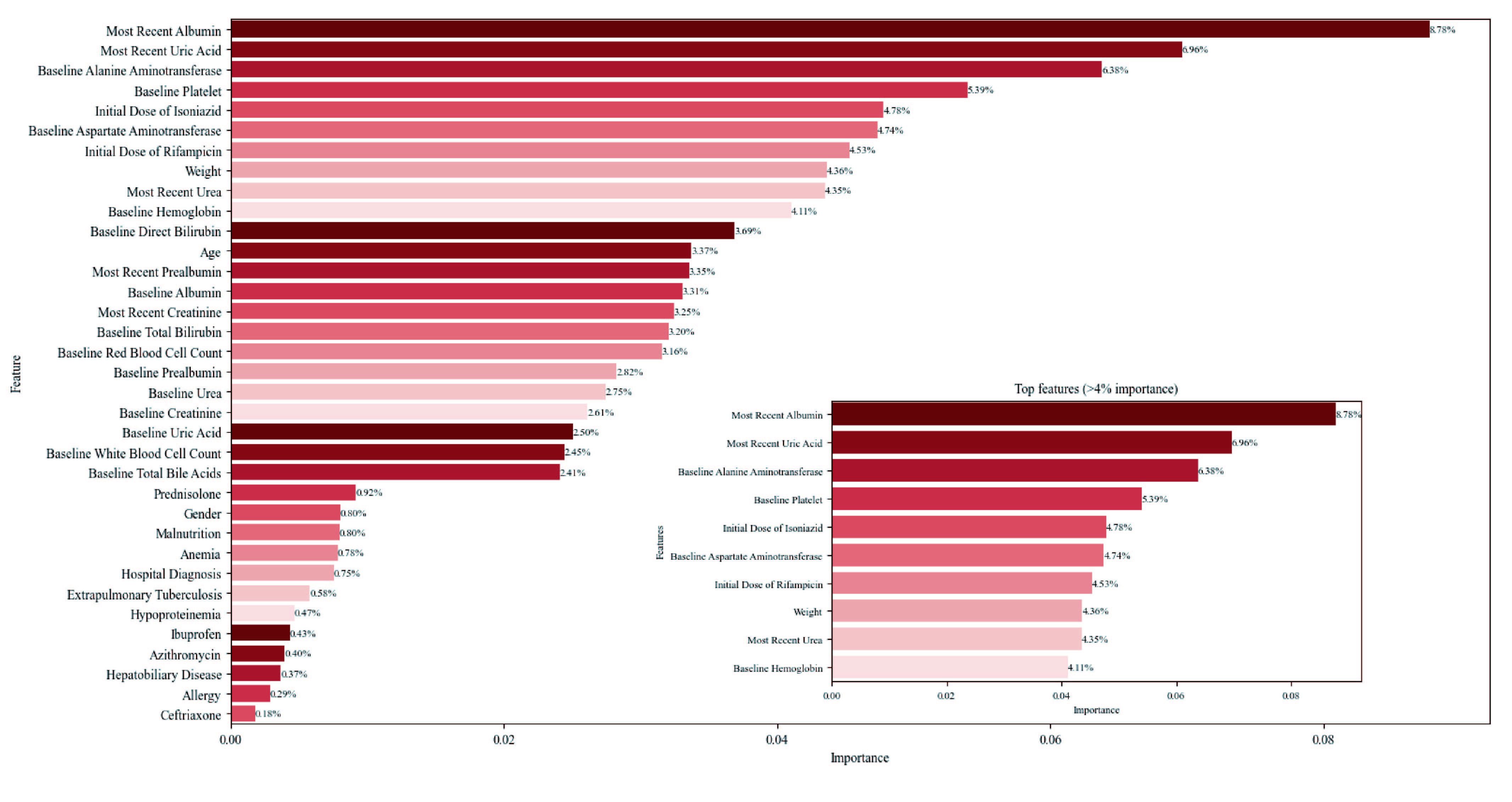

Supplement: Supplementary file 1 [file Image1.jpeg]
